# Molecular Dynamic Study of Mechanism Underlying Nature of Molecular Recognition and the Role of Crosslinker in the Synthesis of Salmeterol-Targeting Molecularly Imprinted Polymer for Analysis of Salmeterol Xinafoate in Biological Fluid

**DOI:** 10.3390/molecules27113619

**Published:** 2022-06-05

**Authors:** Shendi Suryana, Mutakin Mutakin, Yudi Rosandi, Aliya Nur Hasanah

**Affiliations:** 1 Department of Pharmaceutical Analysis and Medicinal Chemistry, Faculty of Pharmacy, Universitas Padjadjaran, Jl. Raya Bandung Sumedang KM 21, Sumedang 45363, Indonesia; shendi@uniga.ac.id (S.S.); mutakin@unpad.ac.id (M.M.); 2 Pharmacy Department, Faculty of Mathematics and Natural Sciences, Universitas Garut, Jl. Jati No. 42B, Garut 44151, Indonesia; 3 Geophysic Department, Faculty of Mathematics and Natural Sciences, Universitas Padjadjaran, Jl. Raya Bandung Sumedang KM 21, Sumedang 45363, Indonesia; rosandi@geophys.unpad.ac.id; 4 Drug Development Study Center, Faculty of Pharmacy, Universitas Padjadjaran, Jl. Raya Bandung Sumedang KM 21, Sumedang 45363, Indonesia

**Keywords:** salmeterol, molecularly imprinted polymer, molecular dynamics, precipitation polymerization

## Abstract

The rational preparation of molecularly imprinted polymers (MIPs) in order to have selective extraction of salmeterol xinafoate (SLX) from serum was studied. SLX is an acting β-adrenergic receptor agonist used in the treatment of asthma and has an athletic performance-enhancing effect. Molecular dynamics were used for the simulation of the SLX-imprinted pre-polymerization system, to determine the stability of the system. The computational simulation showed that SLX as a template, 4-hydroxyethyl methacrylate (HEMA) as a monomer, and trimethylolpropane trimethacrylate (TRIM) as a crosslinker in mol ratio of 1:6:20 had the strongest interaction in terms of the radial distribution functional. To validate the computational result, four polymers were synthesized using the precipitation polymerization method, and MIP with composition and ratio corresponding with the system with the strongest interaction as an MD simulation result showed the best performance, with a recovery of 96.59 ± 2.24% of SLX in spiked serum and 92.25 ± 1.12% when SLX was spiked with another analogue structure. Compared with the standard solid phase extraction sorbent C-18, which had a recovery of 79.11 ± 2.96%, the MIP showed better performance. The harmony between the simulation and experimental results illustrates that the molecular dynamic simulations had a significant role in the study and development of the MIPs for analysis of SLX in biological fluid.

## 1. Introduction

Salmeterol xinafoate (SLX) is a drug classified as a β_2_-agonist, an acting β-adrenergic receptor agonist that is used in the therapy of asthma [1] and has an athletic performance-enhancing effect [2]. β_2_-agonists are on both the World Anti-Doping Agency (WADA) and the International Olympic Committee (IOC) prohibited lists, but salmeterol is allowed in therapeutic doses by inhalation [3]. Inhaled high doses of β_2_-agonists can increase maximal physical activity performance and muscle durability [4], and large doses of β_2_-agonists may have an ergogenic effect; hence, analysis of salmeterol in plasma is required [5]. The concentration of salmeterol in plasma was 0.07 ± 0.03 ng/mL in people with asthma and 0.06 ± 0.03 ng/mL in healthy subjects [3]. The small concentrations of analyte target in complex matrices require selective sample preparation methods [6]. Molecularly imprinted polymers (MIPs) with the ability to recognize molecular targets can be used as novel sorbents for solid phase extraction to extract SLX from biological fluids [7]. MIP is a polymeric porous material with complementary binding features concerning the molecule’s structure, shape, and spatial orientation [8]. As a smart synthetic material, MIP is extensively used in numerous areas including sensors, chromatography, drug delivery systems, and environmental separations/analysis [9]. The application of MIPs is particularly used in solid phase extraction [10,11,12] and, moreover, it is used in analytical columns in chromatography [13,14,15] and as a film layer in sensors [16,17,18]. It is known that the selective recognition of MIP-ligand comes from the interaction between the template and the functional monomer present in the pre-polymerization solution [19]. Therefore, the molecular interaction mechanisms that exist during the pre-polymerization stage can be used as a prognostic tool for polymer performance [20]. Affinity of template molecules can be improved by selecting an appropriate functional monomer. This selection can reduce the non-specific interaction happening in MIPs and enhance the specific recognition of MIPs to the template molecule [21]. The choice of crosslinker is another crucial step in the construction of selective polymer systems [22]. The stability of the interaction between the template and the crosslinker should be weak [23]. The crosslinker may compete with the functional monomer, which may result in the MIPs having poor recognition properties with respect to the targeted molecules [24]. There are some crosslinkers that could be used in the synthesis of MIP, including ethylene glycol dimethacrylate (EGDMA) [25], trimethylolpropane trimethacrylate (TRIM) [26], piperazine diacrylamide (PDA) [27], (+)-*N*,*N*’-diallyltartardiamide (DATD) [28], methylenediacrylamide (MBAA) [29], diisopropenylbenzene [30], bisacryloylamidopyridine [31], *N*,*O*-bismethacryloyl ethanolamine (NOBE) [32], glycidyl methacrylate (GMA) [33], and divinylbenzene (DVB) [34]. Synthesizing MIP relying on experimental trial and error will be very time-consuming and resource-consuming [35]. Nowadays, a computational approach is already used extensively to investigate the design and synthesis of MIP to give MIPs with excellent molecular recognition ability [36].

In a previous study [7], the molecular modeling approach and bulk polymerization method were employed to design and synthesize MIP for the analysis of SLX from spiked serum. A semi-empirical PM3 method was used to calculate the binding energy of the complex between the template–functional monomer and the template–crosslinker, to select the functional monomer with a stable interaction and the crosslinker with the weakest interaction with the template. The calculation results showed HEMA and trimethylolpropane trimethacrylate (TRIM) to be the best functional monomer and crosslinker, respectively. From this calculation, supported by Job plot method experiments, a mol ratio between template:functional monomer of 1:6 was also obtained. To validate the result, the more common ratio (1:4) was used as a comparison. The bulk polymerization method was then used to synthesize the polymers and the agreement between computational and experiment result was established.

To support the molecular modeling result, in the present study, we used molecular dynamics simulations as this is a suitable method for describing the interaction of many components in a system containing a large number of molecules [37]. To improve the binding properties of the previous results [7], we used the precipitation polymerization method to synthesize the novel polymers. The precipitation method was expected to provide a smaller and homogenous particle size with a large surface area that would provide better conditions for optimum binding of SLX to the polymers [38].

## 2. Materials and Methods

### 2.1. Materials

Salmeterol xinafoate (SLX) CAS number 94749-08-3 purity > 98%, terbutaline (TER) CAS number 46719-29-3 purity > 98%, and salbutamol hemisulfate (SAL) CAS number RN 51022-70-9 purity > 98% were purchased from Tokyo Chemical Industry (Tokyo, Japan). 2-Hydroxyethyl methacrylate (HEMA) CAS number 868-77-9 purity 97%, ethylene glycol dimethacrylate (EGDMA) CAS number 97-90-5 purity 98%, trimethylolpropane trimethacrylate (TRIM) CAS number 3290-92-4 purity 98%, and benzoyl peroxide (BPO) CAS number 94-36-0 purity 75% were obtained from Sigma-Aldrich (Singapore). HPLC grade methanol CAS number 47-56-1 purity 99.8%, isopropanol CAS number 67-63-0 purity 99.5%, and acetonitrile CAS number 75-05-8 purity 99.9% were purchased from Fisher Scientific (New York, NY, USA). Acetic acid CAS number 64-19-7 was purchased from Sigma-Aldrich (Singapore). Blood samples were obtained from the Indonesian Red Cross. Empty SPE cartridges were purchased from Supelco (Bellefonte, PA, USA). The Chromabond ABC18 (C18) sorbent solid phase extraction columns were purchased from Macherey-Nagel (Düren, Germany). If not otherwise specified, all chemicals are analytical grade. The morphological evaluation analysis of the polymer was carried out by JSM-6610LV JEOL Ltd. (Tokyo, Japan). BET Surface Area Analyzer Quantachrome NOVA 2200E BET was used in the analysis of the surface area of polymers (Florida, FL, USA). Blood analysis, after extraction with MI-SPE, was performed using HPLC (Waters Alliance e2695 with UV detector) (Florida, FL, USA) with gradient elution, using a water/acetonitrile mixture as the mobile phase. Acetonitrile concentration was changed from 10% to 20% of acetonitrile from minutes 2 to 5, 20% to 50% from minutes 5 to 7, 50% to 10% from minutes 7 to 15. The HPLC column was a Zorbax Eclipse XDB-Column C18 (4.6 × 150 mm, 5 µm) from Agilent Technologies (Santa Clara, CA, USA). The injection volume of the HPLC analysis was 20 µL, using 0.8 mL min^−1^ as the flow rate, and the UV detection wavelength was set at 252 nm. IR analysis was performed using a Nicolet 380 FT-IR from Thermo Fisher Scientific (Waltham, MA, USA). SPE was conducted using SPE manifold purchased from Phenomenex (Torrance, CA, USA).

### 2.2. Molecular Dynamic Simulations

Molecular dynamics simulation was conducted using a molecular system containing one (1) molecule of template, four (4) or six (6) molecules of functional monomer and twenty (20) molecules of crosslinker in a mixed solvent consisting of isopropanol and methanol (1:1). The number of solvent molecules was in accordance with their density. Two-dimensional structures of SLX, HEMA, EGDMA, TRIM, isopropanol, and methanol were drawn using the ChemBio3D Ultra 12.0 program (Developed by CambridgeSoft Corporation, Cambridge, MA, USA) and then converted into three-dimensional structures using the same program. The ab initio method (Hartree Fock, 3–21 G based set), with Games interface on ChemBio3D Ultra 12.0 was used in the geometry optimization step [39]. PACKMOL software (version v20.3.3) [40] was used to pack molecules of the pre-polymerization components randomly within a 40 × 40 × 40 Å box with periodic boundary conditions. Molecular dynamics simulations were conducted using the LAMMPS (version 3 Mar 2020 developed by Sandia National Laboratories, Alburquerque, NM, USA) [41] program combined with reax forcefield [42]. The steepest-descent procedure was used in the energy minimization step with maximum iterations of 200,000. This step is performed to let the molecules rearrange and reach the minimum energy, which occurs during the preparation of the actual pre-polymerization mixture. When the minimum energy was reached, an equilibration step of 0.5 ns was used to first simulate a temperature rise from 0 K to 333 K at atmospheric pressure and, second, a constant high temperature at 333 K at atmospheric pressure. This temperature switch was performed to simulate the temperature shift in the real polymerization processes. The NVT ensembles are used in the simulation of molecular systems, with the number of molecules (N), volume (V), and temperature (T) being kept constant in the system. The 1 ns MD simulation in the explicit solvent was carried out at 333 K, while the coordinates and energy were recovered every 1000 steps, with a step time of 0.1 fs. Calculation of non-bonded interactions was done using a 10.0 Å cut-off. The Ovito program (version 3.7.4 developed by OVITO GmbH, Darmstadt, Germany) was used to visualize and investigate the molecular structure, a 0.05 Å bin size then was employed to calculate the radial distribution function between SLX-HEMA and SLX-crosslinker in the system. 

### 2.3. Synthesis of Molecularly Imprinted Polymer (MIP) of Salmeterol Xinafoate (SLX) and Non-Imprinted Polymer (NIP) Using Precipitation Polymerization

MIP and NIP were synthesized using the precipitation polymerization method, as follows. MIP was obtained by dissolving SLX (1 mmol) as a template and HEMA as a functional monomer (4 mmol / 6 mmol) in 350 mL of an isopropanol:methanol (1:1) mixture, based on constant associations in a previous study [7] in sealed bottles, and then sonicated for 5 min. Subsequently, EGDMA/TRIM (20 mmol) was added to the solution as a crosslinker and then sonicated for 40 min. Benzoyl peroxide (0.206 mmol) was then added to the vial as the initiator, and the vial was finally placed in a water bath shaker at 70 °C for 24 h. The polymer formed was then washed with methanol and water. After washing, the polymer was dried in an oven at 60 °C for 18 h. NIPs were synthesized in the same way, but without the addition of a template, to verify MIP results. The compositions of the synthesized MIP and NIP SLX are presented in Table S1 in Supplementary Materials. The template was removed from the MIP by an ultrasonic extraction method, using 50 mL of a methanol-acetic acid mixture (9:1, *v*/*v*) for 3 h. The polymer was then centrifuged to separate the filtrate. The extracted MIP was then rinsed with 50 mL of the methanol-water mixture (1:1) and dried at 50 °C for 18 h. The same method was used for NIP, to remove the remaining unreacted reagents. To ensure the SLX templates were completely extracted, monitoring was carried out using 10 mg of MIP added to 1 mL of methanol, and the analysis was performed in triplicate. The blank used was 10 mg NIP with 1 mL of methanol. The polymer was then agitated for 3 h at 120 rpm and then set aside. The extraction was complete if there was no more absorbance from the template in the measurement with the microplate. Table S1 shows the composition of precipitation synthesized MIP and NIP in this work, with the same combination as our previous study [7].

### 2.4. Evaluation of MIP and NIP Adsorption Capacity

Evaluation of adsorption capacity was carried out at various concentrations of SLX solution, namely 7.5, 10, and 12.5 ppm. A total of 1 mL of SLX solution of each concentration was added to a vial containing 20 mg of MIP, carried out in triplicate. Twenty micrograms of MIP mixed with 1 mL of solvent without SLX was used as a blank. The vial was then stirred for 3 h at 120 rpm and set aside. The filtrate absorbance was measured using a microplate reader. The data obtained were plotted into Freundlich and Langmuir isotherm adsorption curves. The adsorption capacity evaluation of the NIP was also done with the same procedure as MIP.

### 2.5. Optimization of the MISPE Condition

An empty plastic solid phase extraction cartridge was used for this study. Cartridges with frits at both ends were filled with 200 mg of dry polymer. These are called MISPE and NISPE. Optimization conditions were used to determine the conditioning, loading, washing, and elution solvent that yielded the highest SLX recovery.

$$\mathrm{Recovery}\;=\frac{\mathrm{The}\;\mathrm{area}\;\mathrm{of}\;\mathrm{eluate}}{\mathrm{The}\;\mathrm{area}\;\mathrm{of}\;\mathrm{standard}}\times 100\%.$$

### 2.6. Application of Molecularly Imprinted Solid Phase Extraction (MISPE) and Non-Imprinted Solid Phase Extraction (NISPE) on Spiked Blood Serum

The application of MIP and NIP was carried out on spiked blood serum with SLX standard solution alone and with SLX solution with analogue compounds, namely salbutamol (SAL) and terbutaline (TER). Three-milliliter empty SPE cartridges were filled with 200 mg of MIP and NIP. The SPE process was carried out using a 12-port SPE vacuum manifold from Phenomenex. SPE optimization was carried out with various solvents to obtain optimal conditions during the conditioning, washing, and eluting stages. The optimal conditions were determined from the highest percent recovery obtained through analysis with HPLC. This condition was used further to extract 2 mg/L SLX–spiked blood serum only and with a mixture of SLX with analogue substances. To ensure efficient treatment using MIP, blank serum treated with protein deposition using acetonitrile 3 times the volume of serum and centrifuged for 15 min at 5,000 rpm was injected. All MISPE and NISPE analyses were performed using validated HPLC conditions, with a correlation coefficient for linearity (r) of 0.9995, accuracy 95.81 ± 2.21%, and %RSD for intermediate precision of 1.21%, using acetonitrile:water in gradient condition with 0.8 mL/min flow rate [7].

### 2.7. Physical Characterization of Sorbent with Fourier Transform Infrared (FTIR), Brunauer-Emmett-Teller (BET), and Scanning Electron Microscope (SEM)

The chemical structure of MIP before and after extraction and NIP was characterized by FTIR. Two hundred micrograms of KBr were mixed with 2 mg of sample and then flattened into pellets. Transmission is measured at 400–4000 cm^−1^. The morphology of MIP and NIP sorbents was observed using SEM. The specific surface area of the MIP and NIP was determined using a BET surface analyzer. In the BET method, the specific surface area of the beads is correlated to the amount of N_2_ gas absorbed on the surface of the beads. 

## 3. Results and Discussion

### 3.1. Molecular Dynamic Simulations

In order to have a better insight into the possible interactions and thus an explanation of our results in previous studies [7], we used a molecular dynamic simulation to see the possible interactions in the MIP synthesis pre-polymerization complex. Four molecular dynamics simulations were carried out to describe the effect of the template ratio, functional monomers (T-FMs), and the role of crosslinkers in the stability of T-FMs. MIP1 and MIP3 are systems with a 1:6 mol ratio of T:FMs with different crosslinkers, namely EGDMA or TRIM, respectively, to confirm molecular modeling calculations [7]. The other systems, MIP2 and MIP4, with a 1:4 mol ratio of T-FMs, were used as a comparison to the computational calculations. The 1:4 mol ratio of T-FMs is a more common formula used in making MIP [43]. The mean spatial distribution of all pre-polymerized components was extracted from the trajectories using the radial distribution function (RDF) calculation.

**Figure 1:**
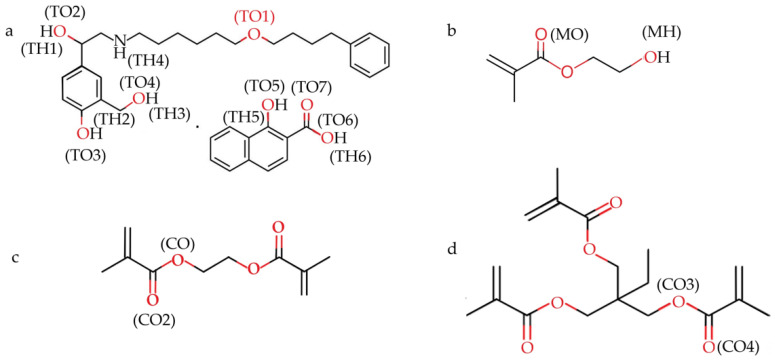
Chemical structure of SLX (**a**), HEMA (**b**), EGDMA (**c**), and TRIM (**d**), and atoms studied with RDFs.

**Figure 2:**
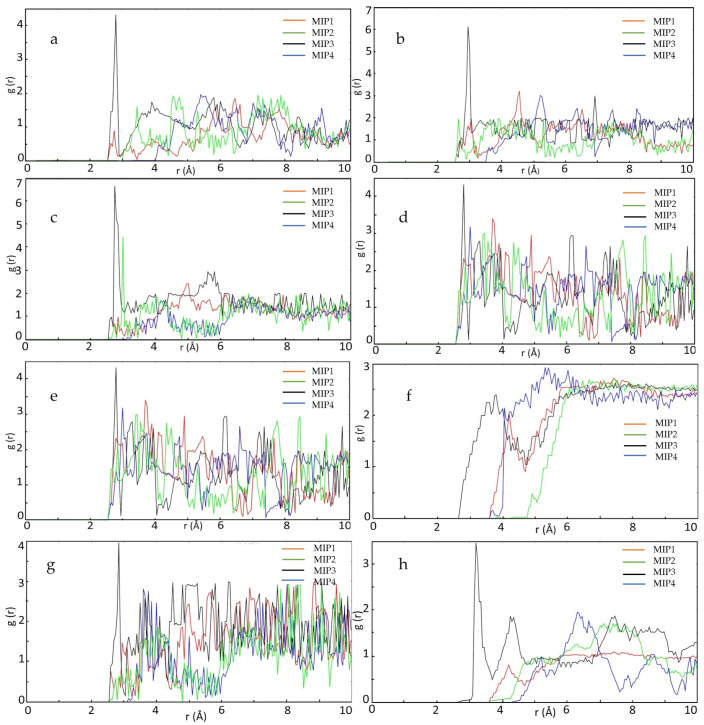
RDF display the probability of SLX oxygen atoms at distinct separation distances from HEMA hydrogen atoms: (**a**) TO1-MH, (**b**) TO2-MH, (**c**) TO3-MH, (**d**) TO4-MH, (**e**) TO5-MH, (**f**) TO6-MH, (**g**) TO7-MH, and (**h**) TH1-MO; r is a certain distance between atoms.

The degree of proximity of SLX to HEMA is discussed from the RDF point of view. In Figure 1, the atoms that can form the interaction site are labeled. The following figure shows RDF atoms that can form interaction sites. This helps predict the state of the HEMA molecule around the SLX. As shown in Figure 2, TO (atom O labeled in SLX)-MH (atom H labeled in HEMA) atomic contact was present. From the result in Figure 2a–f, the distance of hydrogen bonding was between 1.8 and 3.3 Å [44], and MIP3 showed the strongest interaction in each case, with peaks from 3.89 to 6.65. When the distance was more than 3.2 Å, there was no interaction between the TO of SLX and the MH of HEMA. TO3 (the number after “TO” represents the different atom O in SLX) showed the strongest interaction (Figure 2c), which means that the TO3-MH site can play a dominant role in the identification imprinting process. TO6 already has an h-bond with TH4 between SL and X, and the bond interaction between TH4 and TO6 could be stronger than that of HEMA [45], so the RDF of TO6-MH was weaker than the others. 

**Figure 3:**
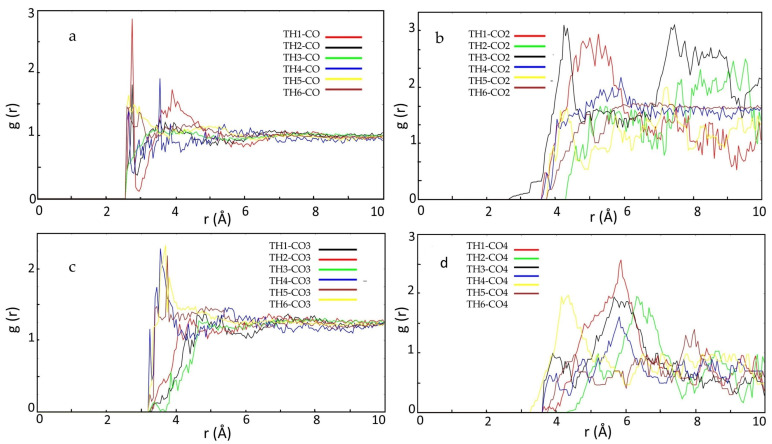
RDFs display the probabilities of hydrogen atoms (TH) of SLX at distinct separation distances from the oxygen atom CO (**a**) and CO2 of EGDMA (**b**), CO3 (**c**) and CO4 of TRIM (**d**).

The effect of the crosslinker on the interaction between SLX with HEMA, between TH (atom H labeled in SLX) and CO (atom C labeled in crosslinker), is depicted in Figure 3. EGDMA showed peaks from 1.5 to 2.86 at a distance of 1.8–3.2 Å, while TRIM did not show this peak in that radius, which means that there is no hydrogen bond between the TRIM and SLX so it will not interfere with the interaction of SLX with HEMA as a functional monomer. This result indicates that the interactions between EGDMA and SLX are stronger than for TRIM. According to this result, TRIM will have a better performance as a crosslinker compared to EDGMA, as it will not affect the binding between SLX and the functional monomer [46].

As shown in Table 1, it is not difficult to see that the strongest site of action was achieved by MIP3 with a T:FM:Cl ratio of 1:6:20 with TRIM as the crosslinker. The van der Waals (VDW) atomic radius of O–H is 2.02–3.02 [47]. The use of the summed VDW radii to determine whether a possible hydrogen bond between the donor and acceptor is suspected is an implicit assumption of atomic interpenetration [48]. The closest distance between the hydrogen bond acceptor and hydrogen bond donor atoms and the density of this distance g(r) indicate that this interaction belongs to the short form and hydrogen bonds are easily formed at the site [49]. Hydrogen bonds generally have an interaction strength of 110 kcal mol^−1^ (4–40 kJ mol^−1^) [50]. The abundance of hydrogen bonds leads to a very stable structure [51]. Thus, the adsorption capacity of MIP3 may be greater than that of MIP 1, MIP2, and MIP4.

### 3.2. Synthesis of the Salmeterol Xinafoate-Imprinted Polymer Using Precipitation Method

The precipitation polymerization method was used to obtain polymers with homogeneous particle sizes. Molecularly imprinted polymer made with precipitation polymerization usually results in spherical particles with diameters less than 1 mm [52]. We used bulk polymerization in a previous study [7], and the results showed weak binding capacity, as seen in the low adsorption capacity. The use of the precipitation method was expected to improve this. The morphology of the polymer that was obtained with the precipitation method was smoother and more homogenous, so the larger surface area of the polymer could interact with SLX, increasing the binding capacity. In our study, the surface area obtained with precipitation was 291.706 m^2^/g for MIP3, while the bulk method gave 221.757 m^2^/g. Bulk polymerization has the following advantages: the compound in the mixture is in a liquid state with no additional solvent, particle size of the resulting MIP is easy to control, it has low cost compared to other methods, and it is easily carried out, whereas the disadvantages are that the obtained MIP requires grinding, there are few deviations in particle shape, and binding sites can be destroyed during the MIP grinding and sieving. Precipitation shows the following advantages: regular shape of MIP beads with good yield, polymer chains grow individually into micro-spheres, porogen agents in the reaction mixture are not needed, the procedure is easy, and it takes less time, while the only drawback is that precipitation occurs when polymer chains are large enough not to dissolve in the reaction mixture [53].

### 3.3. Evaluation of MIP and NIP Adsorption Capacity

**Figure 4:**
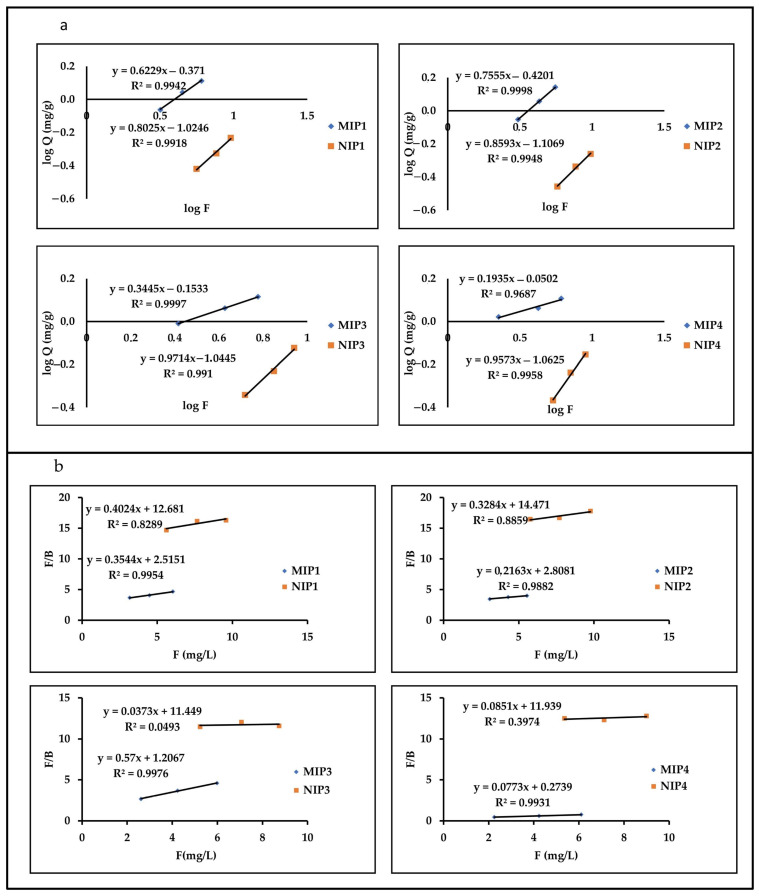
Fitting the adsorption of MIP and NIP to isotherm adsorption model, Freundlich (**a**) and Langmuir (**b**).

The amount of SLX bound to the polymer was determined by batch binding experiments. To describe and determine the adsorption properties of the MIP obtained, the adsorption isotherm data were matched with two types of adsorption isotherm models, namely Langmuir and Freundlich. The Langmuir and Freundlich models are general adsorption isotherm models to describe the adsorption process. For fitting results, we see that the MIP adsorption process is more suitable for the Freundlich model than the Langmuir model; however, NIP follows the opposite pattern. Figure 4 shows the fitting adsorption of MIP and NIP to the adsorption isotherm model.

As shown in Table 2, the suitability of the MIP adsorption to the Freundlich adsorption model showed an R^2^ value above 0.99. NIP is more likely to fit the Langmuir isotherm model with a convincing degree of similarity (R^2^ > 0.99). The difference in the fitting results can be explained by the difference in the adsorption mechanism. For the Freundlich isotherm, the MIP adsorption process involves both surface diffusion and intraparticle diffusion as a result of the interaction of SLX with polymer binding sites; however, NIP adsorption is mainly dependent on surface diffusion [54]. This is also supported by the value of the heterogeneity index (m), which showed that the MIPs had a more heterogeneous distribution of binding sites (near zero) than the NIPs. 

In the previous study with the bulk polymerization method, the adsorption capacity of the polymer was 0.12, 0.05, 0.28, and 0.19 mg/g for MIP1, MIP2, MIP3, and MIP4, respectively. In the present study, the adsorption capacities with the precipitation method were increased, namely 0.46, 0.38, 0.89, and 0.70 mg/g for MIP1, MIP2, MIP3, and MIP4, respectively. This means that the MIPs produced with the precipitation method have adsorption properties that are better than in the previous method [7].

### 3.4. MISPE Optimization

The performance of cartridges packed with MIP particles or NIP particles as sorbents for SLX SPE was investigated. Solid phase extraction (SPE) conditions, such as solution loading, washing, and elution, are carefully optimized for high extraction efficiency. This study tested isopropanol and water to find a suitable loading solvent. Specifically, 1 mL of SLX solution, prepared in the above solvent at a concentration of 2 g/mL, was introduced into the SPE cartridge using 1.0 mL/min flow rate f. Figure 4 shows that only 2.63 ± 0.19% (isopropanol) and 3.11 ± 0.23% (water) template were released into the MIP cartridge (MIP3), whereas 70.53 ± 1.96% (isopropanol) and 54.86 ± 1.16% (water) template were released into the NIP cartridge (NIP3). Therefore, isopropanol and water were chosen as loading solvents.

**Figure 5:**
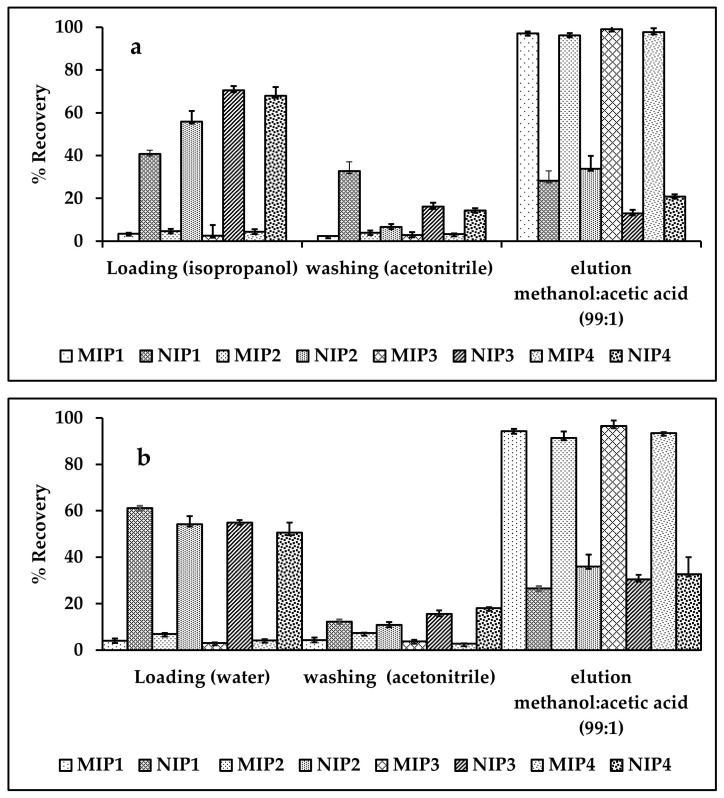
Optimization of MIP and NIP: (**a**) loading with isopropanol, (**b**) loading with water.

In the sample loading process, the template in the sample may be specifically or non-specifically absorbed by the polymer [55]. Therefore, a suitable washing solvent is required to remove the non-specifically bonded mold, while maintaining the specifically bonded mold. Therefore, the solvent used is required to remove the template from the NIP, while maintaining the template in the MIP [56]. In this study, acetonitrile was investigated to optimize washing conditions. The results showed that this effect was obtained by using acetonitrile at a flow rate of 1 mL/min. Figure 5 shows that 2.80 ± 0.42% of SLX was released from MIP3 and 16.09 ± 1.88% from NIP3. The smaller amount of SLX released than MIP indicates that acetonitrile is a suitable washing solvent.

Considering the solubility of SLX and the hydrogen bonding interactions between the template and MIP, a methanol:acetic acid (99:1) mixture was tested to find the optimal elution solution. It was found that the elution of SLX was obtained using 4 × 1 mL of methanol:acetic acid (99:1, *v*/*v*) at a flow rate of 0.5 mL/min. Figure 4 shows that 99.04 ± 0.90% of SLX could be extracted from MIP3 and 13.04 ± 1.59% from NIP3. The higher recovery from the MIP compared to the NIP showed that the elution solvent was suitable.

### 3.5. Effect of Concentration of SLX on % Recovery

**Figure 6:**
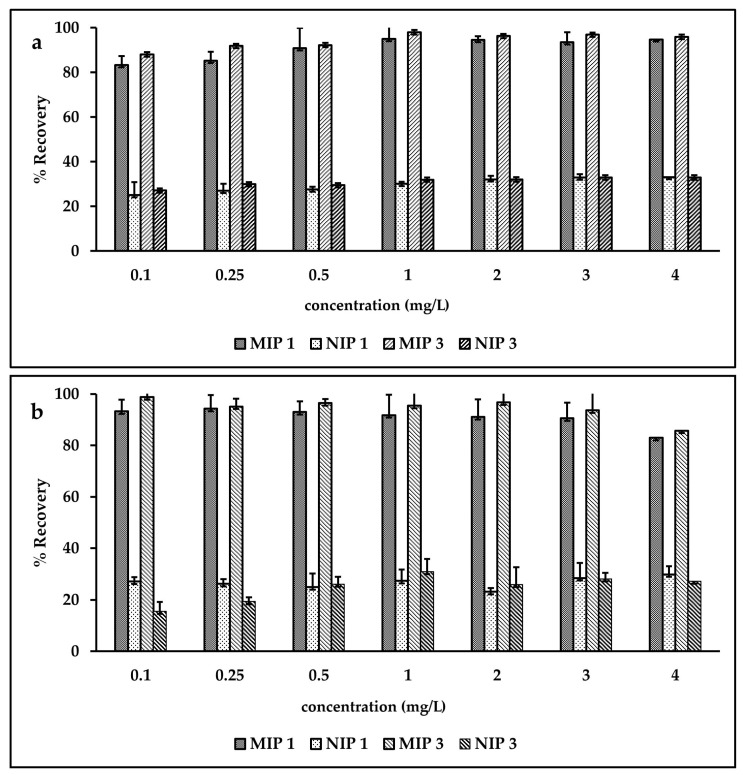
Effect of SLX concentration on % recovery: (**a**) loading using isopropanol, (**b**) loading using water.

To investigate the effect of the concentration of SLX on % recovery, polymers MIP1 and MIP3 were compared to NIP1 and NIP3. These polymers were chosen as being representative of the 1:6 ratio that showed better binding capacities. Ideally, extraction recovery should not rely on sample concentration so there should be no significant difference in recovery across all analyzed concentration ranges [57]. However, this research showed that the recovery of SLX in isopropanol was preferable at a high concentration. The phenomenon that arose upon loading using propanol was concentration dependent because the ability of SLX to produce SLX-SLX complexes is higher in high concentrations, both on the polymer surface and in solution, leading to an increase in the selectivity of SLX [58]. Figure 6 shows that the recovery of SLX with isopropanol as a loading solvent was reduced at low concentration (0.1 µg/mL) compared to high concentration (4 µg/mL). The opposite occurred with water as the loading solvent. The polymer was synthesized to extract SLX in trace concentration; based on the results, water was most suitable for the loading solvent, as it showed a higher recovery in low concentrations than isopropanol. The recovery of SLX at 0.1 mg/L was 87.99 ± 4.57% from MIP3 and 27.10 ± 4.58% from NIP3 with isopropanol as the loading solvent, and 98.75 ± 6.15% from MIP3 and 15.52 ± 3.72 from NIP3 with water as the loading solvent. The higher recovery of SLX on MIP compared with NIP showed that water is better as a loading solvent for the extraction of SLX in trace concentrations. In fact, the concentration of SLX in real plasma is 0.1–2 ng/mL after a single dose (50–400 µg) via inhalation [59]. We believe that loading in water will align with the trace concentrations of SLX in real plasma. 

### 3.6. Selectivity Test

**Figure 7:**
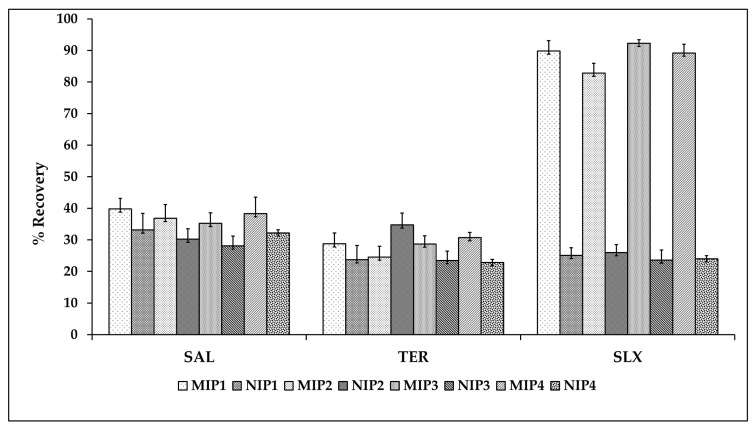
The selectivity of MISPE toward salbutamol (SAL), terbutaline (TER), and salmeterol xinafoate (SLX).

To evaluate the selectivity, two other compounds, i.e., salbutamol (SAL) and terbutaline (TER), were selected. The reason for the choice of these compounds was that they afford many similar functional groups that can bind to MIP. Figure 7 shows that the adsorption recovery of MIP3 for SLX (92.25 ± 1.12%) was much higher than for SAL (35.23 ± 3.34%) and TER (28.71 ± 2.61%). Salbutamol and terbutaline have molecule sizes smaller than salmeterol. The obtained cavity has a larger size for SAL and TER, but in molecular recognition, both functional group and size effects play a part simultaneously. Herein, slight differences between the analogue structure and SLX can affect the mechanism of the “lock–key” recognition rule of the molecular imprinting technique and MIP provides the highest adsorption capacity toward their template [37]. The selectivity result shows that the MIP, especially MIP3, has a higher selectivity to the target of interest than to the other substances, which shows that molecular dynamics simulations combined with the precipitation polymerization method could be used as an approach to fabricate a highly selective polymer as a sorbent to extract SLX from biological matrices in the presence of another similarly structured drug. The present polymers have better selectivity than those of the previous method.

### 3.7. Application of MISPE

**Figure 8:**
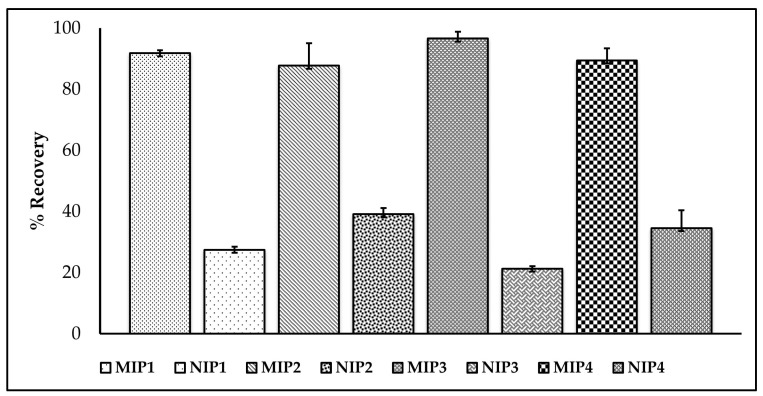
The recoveries of SLX from serum spiked with SLX 2 µg/mL.

To evaluate the optimized MISPE, serum was selected for recovery testing through the standard addition method. The SLX was spiked into the serum at 2 µg/mL. The recovery data, presented in Figure 8, showed that the optimized MISPE could successfully extract SLX from the spiked serum. It should be noted that these recoveries are indicative of the good performance of the MISPE. MIP3 showed the highest recovery (96.59 ± 2.24%) compared to the other MIPs or NIP. This result was better than the previous study with the bulk method (92.17 ± 2.66%) [7] and indicates that MIPs obtained with the precipitation method show better recognition than the other method. This result correlates with the adsorption capacity obtained in the previous stage.

Table 3 shows a comparison of our method to the other study on SLX analysis in blood plasma. The method is comparable to another study on SLX analysis in blood plasma using a more sensitive technology, such as LC-MS/MS assays, as shown in the table. As a result of these findings, we may conclude that our study’s selectivity is comparable to that of the other.

**Figure 9:**
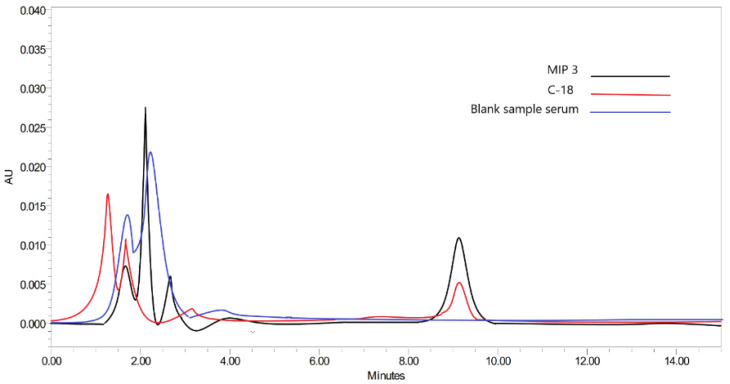
Chromatogram of SLX in serum pre-treated with MIP 3 and C-18 sorbent.

To compare the performances of the synthesized MIP with the available sorbent, the analysis of SLX was also performed using C-18 cartridges as a references [64]. As can be seen in Figure 9, MIP3 (96.594 ± 2.24%) has higher peaks of SLX (retention time 9.28 min) than C-18 (79.11 ± 2.96%), which means that MIP has a better affinity to SLX than C-18 in serum. The recovery of MIP3 from the precipitation method was much higher than that of the previous bulk method (92.17 ± 2.66%) [7], which showed that the present polymers have a better analytical performance than before. 

### 3.8. Physical Characterization of Sorbent with Brunauer-Emmett-Teller (BET), Scanning Electron Microscopy (SEM), and Fourier Transform Infrared (FTIR)

BET analysis (Table 4) shows that the MIPs from the precipitation method have a larger surface area than those of the previous bulk method, which causes the surface capacity for adsorption to be higher and more of the analyte to be adsorbed. Usually, larger surface area indicates better formed particles [65]. The suitable surface area provides the possibility to ensure the correct permeability conditions (good analyte migration through the polymer structure and increasing the probability of reaching the binding site for a given chemical compound) for the sample and organic solvents during analyte collection, as well as the extraction process [66]. The average particle size of bulk particles from our previous paper is 250 µm for MIPs and 428 µm for NIPs, and for precipitation the particle size is 175 µm for MIPs and 224 µm for NIPs [7].

**Figure 10:**
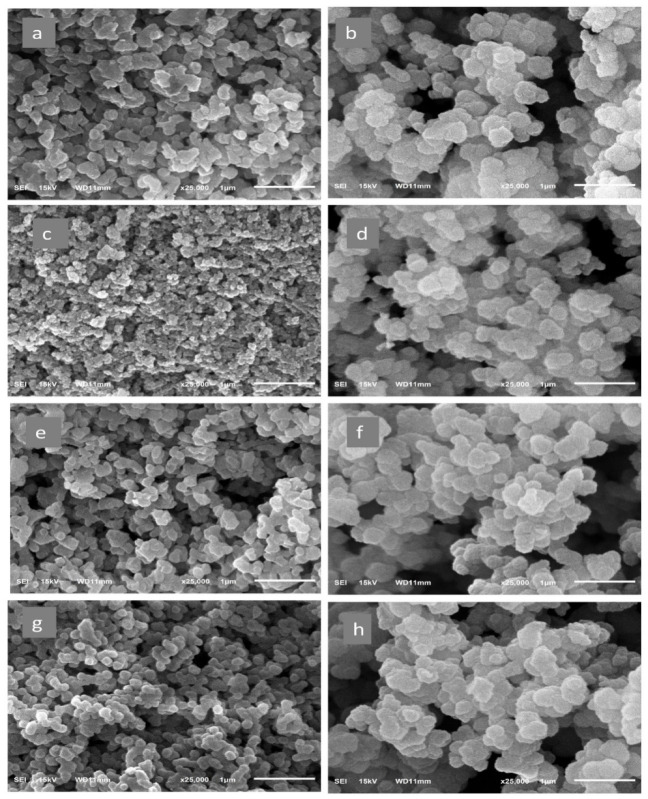
Scanning electron microscope images of (**a**) MIP1, (**b**) NIP1, (**c**) MIP2, (**d**) NIP2, (**e**) MIP3, (**f**) NIP3, (**g**) MIP4, and (**h**) NIP4.

The SEM micrograph (Figure 10) showed that the surface of the MIPs was crude and contained countless pores, but the surface of the NIP was smooth and less porous. The irregular, rough, porous surface of the MIPs is most likely formed by the template removal creating the particular sites of the rebinding cavities [67]. The structural properties of the MIPs indicate a higher adsorption capability [68].

**Figure 11:**
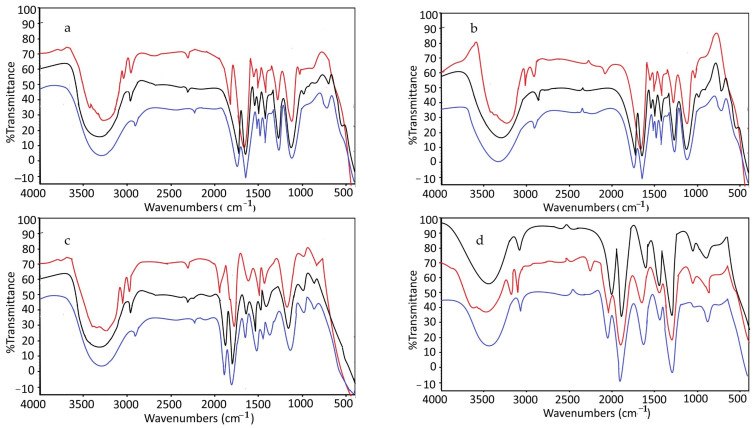
FTIR spectrum of (**a**) MIP1 and NIP1, (**b**) MIP2 and NIP2, (**c**) MIP3 and NIP3, and (**d**) MIP4 and NIP4. Red lines: MIP before extraction, black lines: MIP before extraction, blue lines: NIP.

Figure 11 shows the MIP spectrum before extraction (red line), MIP after extraction (black line), and NIP (blue line). All the polymers, which are MIP1–4, MIP after extraction, and NIP1–4, have a similar backbone and are identical. MIP before extraction has different peaks from wavenumber 3300–3500 cm^−1^ (N–H stretch) and 3010–3100 cm^−1^ (C–H stretch for aromatic benzene ring) indicating the present of SLX, and this peak was not found in MIP after extraction and NIP, indicating the omission of SLX for NIP and the complete removal of SLX as a template in MIP after extraction.

## 4. Conclusions

In this work, MIP was prepared by precipitation polymerization using SLX as the template molecule, HEMA as the functional monomer, and EGDMA and TRIM as crosslinkers. MIP optimized using molecular dynamics simulation according to a template:monomer:crosslinker ratio of 1:6:20 showed stronger interaction from the RDF point of view. No hydrogen bond interaction of TRIM with SLX from the MD result shows that the crosslinker will not affect the binding between template and functional monomer and will result in better molecular recognition with the latter. TRIM-based MIP greatly decreased non-specific adsorption compared to EGDMA-based MIP. The present polymer made with precipitation polymerization had better selectivity over structural analogues and better capacity than in our previous work made with bulk. Moreover, MIP was successfully applied in a solid phase extraction in serum analysis and effectively extracted SLX from the complex serum matrix, confirming the elimination of interference and achieving high SLX recovery.

## Figures and Tables

**Table 1:** RDF of oxygen atoms of SLX with hydrogen of HEMA in range of hydrogen bonding radii.

Polymer	TO1-MH		TO2-MH		TO3-MH		TO4-MH		TO5-MH		TO6-MH		TO7-MH	
	r (Å)	g (r)	r (Å)	g (r)	r (Å)	g (r)	r (Å)	g (r)	r (Å)	g (r)	r (Å)	g (r)	r (Å)	g (r)
MIP1	2.7386	0.887	3.040	1.957	2.889	0.925	3.1909	2.737	2.7386	2.324	-	-	2.6884	0.783
MIP2	-	-	-	-	3.1407	0.083	2.7889	0.7708	3.1909	3.438	-	-	3.1909	0.316
MIP3	2.7885	4.328	2.939	6.125	2.7386	6.658	3.1407	4.863	2.7889	4,386	3.2	0.175	2.7386	3.895
MIP4	3.1407	0.486	2.638	1.951	2.9899	4.449	3.0904	2.9624	2.839	2.295	-	-	2.6381	0.675

**Table 2:** Isotherm adsorption of MIP and NIP.

Polymer	Langmuir			Freundlich		
	R^2^	K_L_ (L/mg)	Q_m_ (mg/g)	R^2^	m	a (mg/g)
MIP 1	0.9954	0.1409	2.8216	0.9998	0.5691	0.4640
NIP 1	0.8289	0.0317	2.4850	0.9918	0.8025	0.0944
MIP 2	0.9882	0.0770	4.6232	0.9994	0.7555	0.3801
NIP 2	0.8859	0.0226	3.0450	0.9948	0.8593	0.0781
MIP 3	0.9997	0.2721	3.6751	0.9997	0.2721	0.8908
NIP 3	0.9958	0.9573	1.0446	0.9958	0.9573	0.0865
MIP 4	0.999	0.346	2.8901	0.999	0.346	0.7025
NIP 4	0.991	0.9714	1.0294	0.991	0.9714	0.0902

**Table 3:** Comparison of repeatability, recovery, and LOQ values with other research studies.

Target	Preparation Sample	Instrument	Repeatability	% Recovery	Limit of Quantification	Reference
Salmeterol	SPE	HPLC	3.7–16.3%	74–84%	2.0 ng/ml	[60]
Salmeterol xinafoate	SPE	LC-MS/MS	1.86–6.12%	98.31–100.00%	2.0 pg/mL	[61]
Salmeterol xinafoate	LLE	HPLC	>2.2%	98.2–102.7%	0.025 μg/mL^−1^	[62]
Salmeterol xinafoate	LLME	HPLC	6.0–8.5%	>90.0%	0.30 ng/ mL	[63]
Salmeterol xinafoate	LLE	LC-MS	8.8–13.7%	103.6%	2.5 pg/mL	[64]
Salmeterol xinafoate	MISPE	HPLC	1.7–6.8%	98.7%	6.3 ng/ml	Our result

**Table 4:** Surface area of MIP and NIP.

Polymer	Surface Area (m^2^/g)	
	Precipitation Method	Bulk Method [7]
MIP1	254.419	42.297
NIP1	23.051	18.367
MIP2	211.486	40.674
NIP2	35.393	6.033
MIP3	291.706	221.757
NIP3	40.216	61.381
MIP4	230.160	141.370
NIP4	32.882	37.131

## Data Availability

Not applicable.

## Supplementary Materials

The following supporting information can be downloaded at: https://www.mdpi.com/article/10.3390/molecules27113619/s1. Table S1: The Compositions of synthesized MIPs and NIPs using precipitation polymerization.

## Abbreviations

SLX	Salmeterol xinafoate
WADA	World Anti-Doping Agency
IOC	International Olympic Committee
MIPs	Molecularly imprinted polymers
TER	Terbutaline
SAL	Salbutamol
HEMA	Hydroxyethyl methacrylate
EGDMA	Ethylene glycol dimethacrylate
TRIM	Trimethylolpropane trimethacrylate
BPO	Benzoyl peroxide
NIPs	Non-imprinted polymers
MISPE	Molecularly imprinted solid phase extraction
NISPE	Non-imprinted solid phase extraction
HPLC	High-performance liquid chromatography
FTIR	Fourier transform infrared
SEM	Scanning electron microscope
BET	Brunauer-Emmett-Teller
T	Template
FM	Functional monomer
RDF	Radial distribution factor
VDW	Van der Waals
