# Supplementary material for: Molecular Dynamic Study of Mechanism Underlying Nature of Molecular Recognition and the Role of Crosslinker in the Synthesis of Salmeterol-Targeting Molecularly Imprinted Polymer for Analysis of Salmeterol Xinafoate in Biological Fluid
Source: Molecules. 2022 Jun 5;27(11):3619. doi: 10.3390/molecules27113619 (PMC9182462; doi:10.3390/molecules27113619)
Supplement: Supplementary file 1 [file molecules-27-03619-s001.zip › molecules-1726603-supplementary.pdf]

Supplementary Material

Table S1. The Compositions of synthesized MIPs and NIPs using precipitation polymerization.

Polymers	Template (T)	Functional Monomer (FM)	Crosslinker (Cl)	Ratio T:FM:Cl(mol)
MIP1	SLX	HEMA	EGDMA	1:6:20
NIP1	-	HEMA	EGDMA	1:6:20
MIP2	SLX	HEMA	EGDMA	1:4:20
NIP2	-	HEMA	EGDMA	1:4:20
MIP3	SLX	HEMA	TRIM	1:6:20
NIP3	-	HEMA	TRIM	1:6:20
MIP4	SLX	HEMA	TRIM	1:4:20
NIP4	-	HEMA	TRIM	1:4:20
